# Phytoremediation Potential of Heavy Metals Using Biochar and Accumulator Plants: A Sustainable Approach Towards Cleaner Environments

**DOI:** 10.3390/plants14223470

**Published:** 2025-11-14

**Authors:** Marcos Rosas-Ramírez, Efraín Tovar-Sánchez, Alexis Rodríguez, María Luisa Castrejón-Godínez, Hugo Albeiro Saldarriaga-Noreña, Luz Bretón-Deval, Patricia Mussali-Galante

**Affiliations:** 1 Centro de Investigación en Biotecnología, Universidad Autónoma del Estado de Morelos, Av. Universidad 1001, Col. Chamilpa, Cuernavaca C.P. 62209, Morelos, Mexico; marcos.rosas@uaem.edu.mx (M.R.-R.); alexis.rodriguez@uaem.mx (A.R.); 2 Centro de Investigación en Biodiversidad y Conservación, Universidad Autónoma del Estado de Morelos, Av. Universidad 1001, Col. Chamilpa, Cuernavaca C.P. 62209, Morelos, Mexico; efrain_tovar@uaem.mx; 3 Facultad de Ciencias Biológicas, Universidad Autónoma del Estado de Morelos, Av. Universidad 1001, Col. Chamilpa, Cuernavaca C.P. 62209, Morelos, Mexico; mlcastrejon@uaem.mx; 4 Centro de Investigaciones Químicas, Universidad Autónoma del Estado de Morelos, Av. Universidad 1001, Col. Chamilpa, Cuernavaca C.P. 62209, Morelos, Mexico; hsaldarriaga@uaem.mx; 5 Instituto de Biotecnología, Universidad Nacional Autónoma de México, Av. Universidad 2001, Col. Chamilpa, Cuernavaca C.P. 62210, Morelos, Mexico; lzbreton@ibt.unam.mx

**Keywords:** mine tailing, pyrolysis, genotoxic damage, coconut fiber, bioconcentration factor

## Abstract

Native plant species show significant promise for the remediation and rehabilitation of mine tailings contaminated with heavy metals (HM). Nonetheless, the harmful impact of HM can decrease plant survival, growth and reproduction, thereby diminishing the effectiveness of phytoremediation. Consequently, incorporating organic amendments into mine tailings, like biochar, can promote plant growth, decreasing the bioavailability of HM and their eventual potential to alter the food chain. This study aims to evaluate the capability of coconut fiber biochar in combination with *Sanvitalia procumbens* to phytostabilize HM in mine tailings by analyzing the effect of coconut fiber biochar on HM bioaccumulation levels (roots and leaves), as well as on morphological, physiological, and genotoxic parameters of *S. procumbens* grown in mine tailing substrate and mine tailing/biochar. Also, a physicochemical analysis of coconut fiber biochar was conducted. This research was conducted over 100 days on plants grown in greenhouse settings using two different substrates (mine tailing and agrolite [75/25 *v*/*v*] and mine tailing and coconut fiber biochar [75/25 *v*/*v*]). Every 25 days, 12 plants were selected per treatment for analysis. The bioaccumulation pattern exhibited by *S. procumbens* was Zn > Pb > Cu > Cd, in root and leaf tissues for both treatments. *S*. *procumbes* grown in mine tailing/biochar substrate showed the lowest HM bioaccumulation levels in both tissues in comparison to mine tailing substrate: Zn from 2.95 to 2.50 times lower; Pb 3.04 to 2.82; Cu 3.10 to 2.12; and Zn 2.12 to 3.00 in roots and leaves, respectively. The coconut fiber biochar was rich in functional groups, such as carboxyl and hydroxyl groups, which could favor HM adsorption. Immobilization percentage of HM by coconut fiber biochar showed the following pattern: Pb (66.33%) > Zn (64.50%) > Cu (62.82%) > Cd (55.39%). Incorporating coconut fiber biochar as an amendment improves HM phytostabilization efficiency by reducing their bioaccumulation, increasing biomass production and chlorophyll concentration, and reducing genetic damage levels. This strategy represents a sustainable approach towards reducing the ecological risk of HM biomagnification, alleviating the adverse effects of HM exposure on ecosystem health.

## 1. Introduction

Various remediation techniques have been developed to contain, clean up, mitigate, or restore heavy metal (HM) contaminated soils, but their effectiveness and costs vary significantly in field practice. Among promising soil remediation techniques are electrokinetic extraction, chemical stabilization, and bioremediation [1]. Bioremediation uses microorganisms, fungi, plants, or their metabolites to restore the natural environment altered by contaminants to its original condition. A key example of bioremediation is phytoremediation, where plants are employed to retain, eliminate, or diminish contaminants in soils [2]. This technique has proven efficient because it can be applied in situ, it keeps the biological properties and physical structure of soils, and its costs are 10 to 100 times lower than physicochemical processes [3]. Anthropogenic activities like metal mining contribute the most to soil pollution through the generation of mine tailings, which contain high concentrations of toxic HM, which are not biodegradable, bioaccumulate in exposed individuals, and present high environmental persistence, which represents an important environmental and human health risk [4]. Hence, it is important to improve phytoremediation systems to reduce the bioavailability, mobility, and translocation of HM to the aerial parts of the plants in order to prevent their biomagnification through the food chain. In this context, plants that grow in HM-polluted soils develop adaptive and survival strategies adequate for growing on soils with low availability of nutrients and water, high HM concentrations, adverse soil conditions (acid soils), and compacted substrates; these characteristics along with particular plant traits such as short life cycles, wide geographic distribution, massive root system, high biomass production, and easy harvesting are key elements of HM accumulator plants that are employed for phytoremediation [5]. Although good results have been reported for HM phytoremediation, this process can take a long time, and plant growth and survival may be compromised. To overcome this, the use of accumulator plants in combination with organic amendments (i.e., composts, manure, biosolids, and biochar) is used for the remediation of soils polluted with inorganic contaminants, like HMs. This kind of approach is known as assisted phytoremediation. It has been reported that the use of amendments to increase plant growth and development, in addition to improving the physicochemical properties of soils polluted with mining waste, can enhance the efficiency of phytoremediation, specifically the phytostabilization of HMs in soils [6].

Therefore, the application of biochar (BC) can be a highly effective alternative for enhancing conventional phytoremediation techniques, as it is a porous carbonaceous material obtained from the thermal degradation of organic materials in the absence of or limited oxygen (pyrolysis) [7]. Various raw materials have been used for its production [8]. In this study, coconut fiber was used as a raw material to produce BC since the coconut plant (*Cocos nucifera*) is one of the most popular crops in tropical regions and is economically important in many countries [9], with Mexico being among the top ten countries in coconut production [10]. Furthermore, due to the multiple uses of the coconut fruit, high amounts of waste are generated, with poor management and handling, becoming an environmental problem. Moreover, their disposal is done through open-air burning, producing environmental pollutants such as greenhouse gases, promoting global warming [11]. Hence, using these agro-industrial wastes to produce BC is a sustainable way to create amendments useful for assisted phytoremediation strategies. Moreover, BC produced from organic waste has gained relevance worldwide since it helps in carbon sequestration, improving soil fertility, energy production, and environmental remediation [12,13,14,15], due to its use as a universal adsorbent of organic and inorganic pollutants.

HM bioaccumulation in plants results in negative effects on physiological, biochemical, and molecular processes, which in turn lead to significant growth and developmental alterations [16,17]. These detrimental effects may affect the efficiency of phytoremediation. Hence, it is crucial to evaluate the damage caused by HMs. Different biomarkers of effect have been employed, such as morphological (biomass production), physiological (chlorophyll content), and genetic (genotoxic damage) traits, which in turn, provide us with relevant information on the plant’s overall health and how BC can influence these plants’ responses to chemical stress.

*Sanvitalia procumbens* is an herbaceous plant belonging to the Asteraceae family. This species can bioaccumulate HMs such as Cd, Pb, and Zn in roots and leaves [18,19]. It exhibits rapid growth (5–7 months) with a massive root system that develops close to the surface, which is attributed to this particular trait [18]. It also has a wide geographical distribution (Central and North America, Europe, and Asia) and grows in different climates (warm, semi-warm, semi-dry, dry, and temperate), mainly in disturbed environments with nutrient deficiencies, facts that demonstrate its high phytoremediation potential. Generally, phytoremediation studies are carried out either with plants [20,21] or with biochar [22,23], but the combination of plant and biochar is rarely used [24,25,26]. This study is novel in its approach to valorizing agro-industrial waste derived from coconut fiber, which can be highly polluting when not properly managed. Coconut fiber biomass was converted into biochar to facilitate the immobilization of HM present in the soil, while simultaneously enriching the soil with nutrients that promote the establishment of the HM accumulator species *S*. *procumbens*. The application of this binomial could be an effective strategy for the phytoremediation of HM-contaminated soils across diverse types of vegetation, given the wide geographic distribution of this species.

Hence, the present study aims to evaluate the capability of coconut fiber BC in combination with *S*. *procumbens* to phytostabilize HM in mine tailings and to analyze the effect of coconut fiber BC on HM bioaccumulation levels (roots and leaves), as well as on morphological, physiological, and genotoxic parameters of *S. procumbens* grown on mine tailing substrate.

## 2. Results

### 2.1. Physicochemical Characterization and Heavy Metal Content in Coconut Fiber Biochar

According to the physicochemical characterization, coconut fiber biochar exhibited a pH of 10, with 4.4% OM, 10.5 mg·kg^−1^ phosphorus, 0.33 mg·kg^−1^ total nitrogen, 2.6 (%), organic carbon, and a C/N ratio of 78. Based on the reference values established by the Mexican standard NOM-021-SEMARNAT-2000, coconut fiber biochar was classified as a strongly alkaline substrate with a high proportion of organic matter. In contrast, both the C/N ratio and phosphorus content fell within the intermediate range. No other metal was detected, except for Zn (35 ± 5 mg·kg^−1^).

The carbonaceous material obtained (biochar) showed an alkaline pH of 10, indicating the presence of basic groups on the surface, possibly associated with metal hydroxide residues or partially oxidized graphitic structures [27]. The organic matter (OM) content was 4.4%, while the organic carbon accounted for 2.6%, thus suggesting that most of the carbon in the biochar samples is structured in inorganic forms or in non-oxidized aromatic structures [28]. The total nitrogen concentration was 0.33 mg·kg^−1^, and the phosphorus concentration was 10 mg·kg^−1^, suggesting traces of residual nutrients from the plant precursor [29,30].

The elemental composition of the coconut fiber biochar, as determined through FE-SEM, revealed a high content of carbon (80.26%) and oxygen (18.73%), confirming the predominance of a carbonaceous matrix with a surface functionalized with oxygenated groups. Trace elements detected included Ca (0.78%), Cl (0.27%), K (0.17%), Si (0.14%), and Na (0.09%), typical of heat-treated lignocellulosic materials, while the elements Al, Mg, S, and Cr were present at concentrations below 0.1% [29,31].

Organic elemental analysis revealed that the material contains 75.08% carbon, a low proportion of hydrogen (0.44%), and an absence of nitrogen and sulfur, indicating high carbon aromatization with little nitrogen or sulfur functionalization [32]. Finally, the material showed a production yield (D.W.) of 31.58%, which is consistent with reports for biochar obtained from lignocellulosic waste [32].

### 2.2. Coconut Fiber Biochar FTIR Analysis

The FTIR spectrum obtained for coconut fiber biochar revealed the presence of various surface functional groups, reflecting the structural residues of the original lignocellulosic components, and those generated by the pyrolysis process of the coconut fiber. As expected, the bands corresponding to aliphatic C-H bonds (2700 and 2900 cm^−1^) were absent in the biochar sample. Moreover, the absence of a broad band around 3400–4500 cm^−1^ corresponding to vibrational stretching of –OH bonds indicates that the hydroxyl groups from both cellulose and lignin were eliminated as a result of the biochar production process.

The signals located around 1550 cm^−1^ are attributed to C=C stretching of aromatic rings, demonstrating the presence of condensed aromatic structures associated with the thermal degradation of lignin. The band located at 1100 cm^−1^ corresponds to C−O bonds characteristic of esters, alcohols, or ethers originating from cellulose and hemicellulose present in the original raw material. The bands observed at 850 and 950 cm^−1^ correspond to C–H bonds in aromatic structures. Finally, the strong band in the 500–600 cm^−1^ region could indicate out-of-plane deformations of C–H bonds in aromatic structures, consistent with partial lignin carbonization.

These results suggest that, although the activation process eliminates a large portion of volatile compounds, functional fragments of lignocellulosic origin are still retained and may contribute significantly to adsorption mechanisms through hydrophobic interactions, hydrogen bonding, and π–π stacking [33,34,35] (Figure S1).

### 2.3. Thermogravimetric Analysis (TGA) of the Coconut Fiber Biochar

According to the profile of the thermogravimetric analysis (TGA), two thermal events were observed (Figure S2). The first observed mass loss was around 11.09% in the temperature interval of 20–400 °C, this mass loss could be related to the elimination of water joined to the biochar through physical adsorption, and the decomposition of oxygenated functional groups, i.e., hydroxyl (–OH) and carboxyl (–COOH), distributed at the surface of this material, as previously reported for biochar produced from agricultural residues [36,37]. The second mass loss (14.73%), observed between 400 and 590 °C, could be associated with the degradation of carbonaceous structures with low stability and partial oxidation of residual graphitic moieties [38]. These results indicate that coconut fiber biochar has a moderate thermal stability, maintaining more than 70% of its mass up to 600 °C, which is characteristic of biochar generated with residual biomass [39]. Furthermore, the initial loss indicates a surface rich in functional groups, which could favor adsorption processes through polar, hydrophilic, or acid-base interactions [40].

### 2.4. Heavy Metal Bioaccumulation in Roots and Leaves of S. procumbens Growing on Tailing/Tailing-Biochar Substrates

#### 2.4.1. Roots

**Figure 1:**
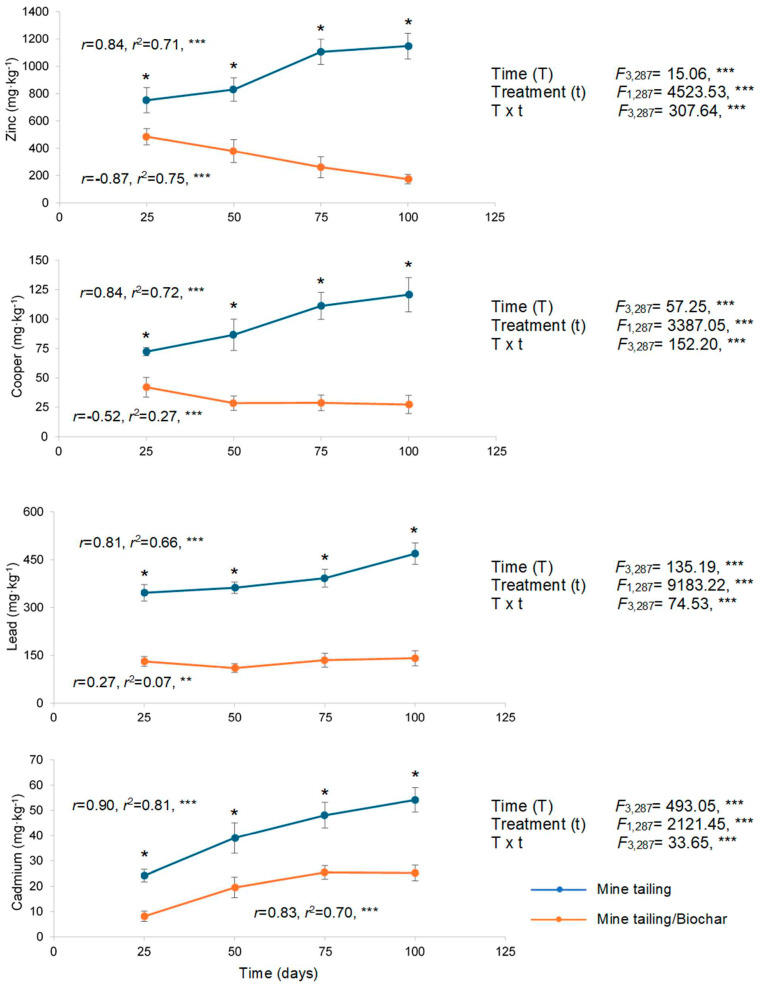
Heavy metal concentration (mean ± standard deviation), two-way ANOVA assessing the effects of treatment, time, and their interaction (time × treatment) in roots of *S. procumbens* grown under greenhouse conditions. Regression analysis between exposure time and heavy metal concentration in roots. Asterisks indicate significant differences between treatments at each exposure time (* *p* < 0.05, Tukey test). ANOVA test: ** = *p* < 0.01, *** = *p* < 0.001.

Our results showed that *S. procumbens* accumulated Cd, Cu, Pb, and Zn in roots. The bioaccumulation potential of this species was significantly influenced by time, treatment, and their interaction (time × treatment) (Figure 1). Across all exposure times, plants grown in the tailing/biochar substrate showed significantly lower concentrations of all analyzed metals compared to those grown in tailings (Tukey test).

Regression analyses indicated that in the roots of *S. procumbens* individuals growing in tailing substrate, metal bioaccumulation increased as exposure time also increased. In contrast, in the tailing/biochar treatment, Pb and Cd exhibited positive time-dependent accumulation, whereas Cu and Zn concentrations declined significantly with prolonged exposure (Figure 1).

The pattern registered for the mean metal bioaccumulation in roots was Zn > Pb > Cu > Cd for both substrates; however, concentrations (mg·kg^−1^) were substantially lower for individuals growing in tailing/biochar (Zn: 324.75; Pb: 129.25; Cu: 31.50; Cd: 19.50 mg·kg^−1^) compared with tailing substrate (Zn: 958.50; Pb: 392.50; Cu: 97.50; Cd: 41.25 mg·kg^−1^). Also, the next pattern was identified regarding metal immobilization when coconut biochar was added to the substrate: Cu (67.7%) > Pb (67.1%) > Zn (66.1%) > Cd (52.7%).

#### 2.4.2. Leaves

**Figure 2:**
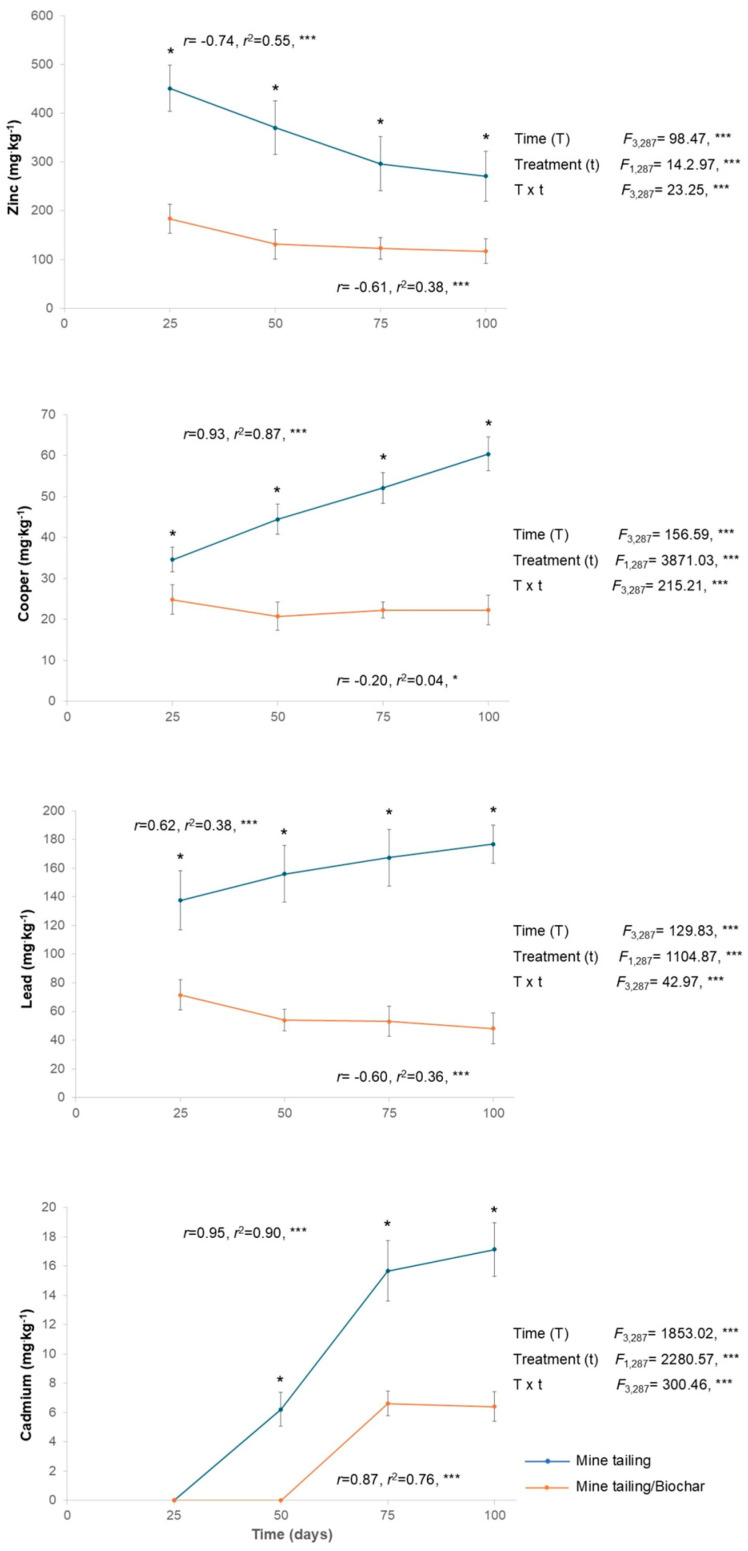
Heavy metal concentration (mean ± standard deviation), two-way ANOVA assessing the effects of treatment, time, and interaction (time × treatment) in leaves of *S. procumbens* grown under greenhouse conditions. Regression analysis between exposure time and heavy metal concentration in leaves. Asterisks indicate significant differences between treatments at each exposure time (* *p* < 0.05, Tukey test). ANOVA test: *** = *p* < 0.001.

In general, metal bioaccumulation in the leaves of *S. procumbens* (Cd, Cu, Pb, and Zn) was registered. Specifically, we observed a significant effect of time, treatment, and the interaction (time × treatment) on the bioaccumulation of all the analyzed metals (Figure 2). Statistically significant differences between treatments were observed at all exposure times, with plants grown in the tailing/biochar substrate consistently exhibiting the lowest levels of metal bioaccumulation compared with those grown in tailing substrate (Figure 2).

For *S. procumbens* individuals growing in tailing substrate, a positive and significant relationship was found between exposure time and the bioaccumulation of all analyzed metals, except for Zn, which displayed a significant and negative relationship. In contrast, for plants grown in tailing/biochar, only Cd showed a positive and significant relationship with exposure time, whereas Cu, Pb, and Zn exhibited significant and negative relationships.

Moreover, plants grown in both substrates displayed the following mean bioaccumulation pattern (mg·kg^−1^): Zn (347.25 and 138.75) > Pb (159.50 and 56.61) > Cu (47.75 and 22.50) > Cd (9.75 and 3.25) in tailing and tailing/biochar, respectively.

### 2.5. Bioconcentration and Translocation Factors of S. procumbens Grown in Tailing and Tailing/Biochar Substrates

The bioconcentration factor (BCF) found was Pb > Cu > Cd > Zn in both roots and leaves of individuals grown in tailings and in tailing/biochar substrates. Overall, BCF values were significantly lower in plants grown in the tailing/biochar substrate compared with those in the tailing substrate (Table 1). The translocation factor (TF) was <1 under both treatments (Cu > Zn > Pb > Cd), indicating that plants growing in tailings exhibited significantly lower TF values for Cu, Pb, and Zn than those grown in tailing/biochar. In contrast, plants cultivated in tailing/biochar showed significantly lower TF values for Cd compared with those grown in tailing substrate (Table 1).

### 2.6. Effect of Tailing and Tailing/Biochar Substrates on S. procumbens Biomass

**Figure 3:**
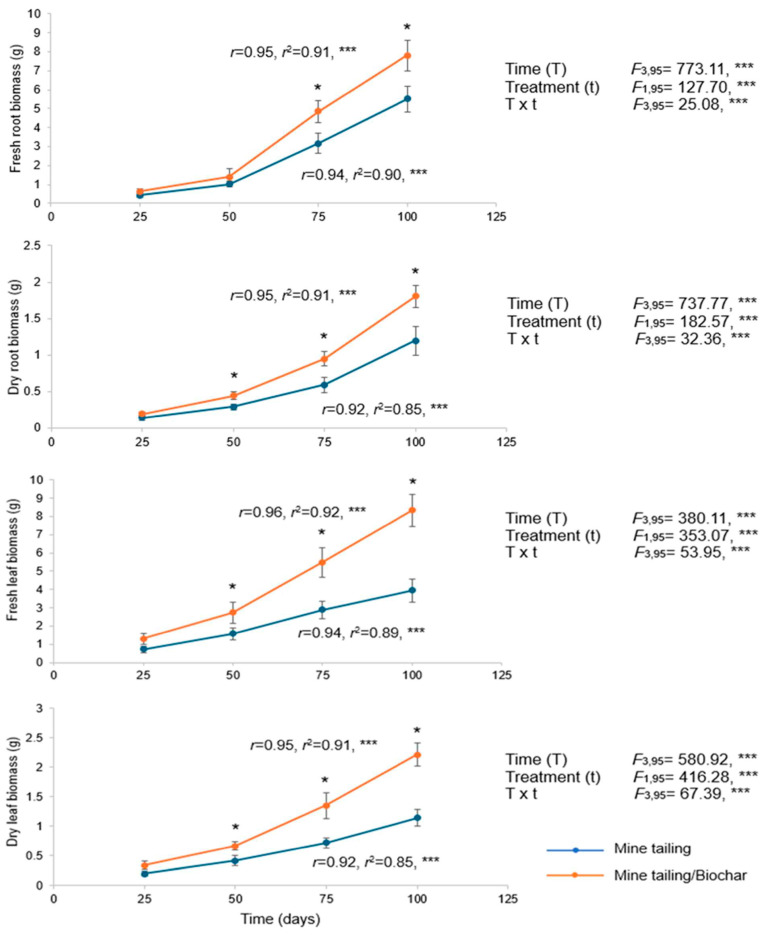
Biomass (mean ± standard deviation) of roots and leaves of *S. procumbens* grown under greenhouse conditions in tailing and tailing/biochar substrates. A two-way ANOVA was performed to evaluate the effects of time (100 days) and treatment on root and leaf biomass traits. Simple regression analysis was used to assess the relationship between exposure time and biomass traits. Asterisks indicate significant differences between treatments at each exposure time (* *p* < 0.05, Tukey test). ANOVA test: *** = *p* < 0.001.

The results of this study showed a significant effect of time, treatment, and their interaction (time × treatment) on *S. procumbens* biomass, including wet and dry root and leaf biomass (Figure 3). Overall, from day 50 of exposure onward, plants grown in the tailing/biochar substrate exhibited significantly higher biomass compared with those grown in the tailing substrate. Regression analyses revealed a positive and significant relationship between exposure time and all measured biomass traits, indicating that as exposure time increased, biomass traits also increased in *S. procumbens* plants (Figure 3).

### 2.7. Effect of Tailing and Tailing/Biochar Substrates on Chlorophyll a and b Content of S. procumbens Individuals

The results showed a significant effect of time, treatment, and the interaction (time × treatment) on the concentration of chlorophyll *a* and *b* in *S. procumbens*. Chlorophyll *a* and *b* concentrations decreased over time in all plants, with significant differences between treatments at each exposure period. Plants grown in the tailing/biochar substrate exhibited significantly higher chlorophyll a and b contents compared with those grown in tailings alone.

**Figure 4:**
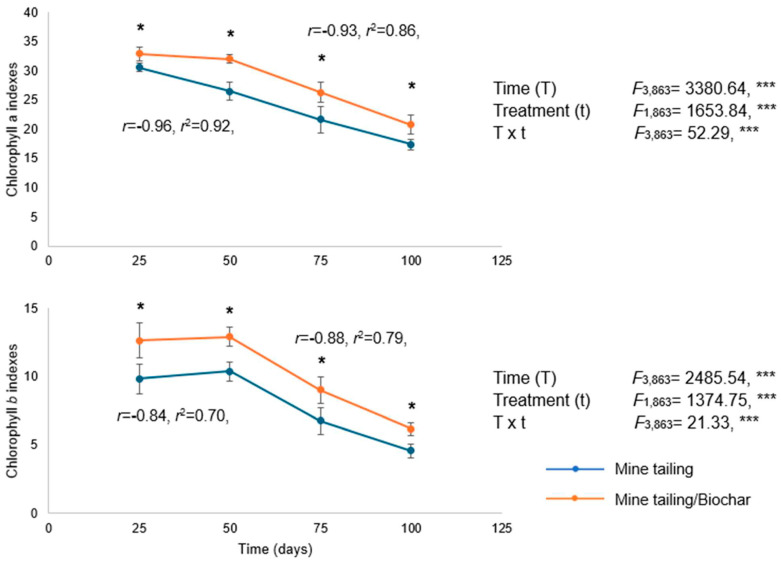
Chlorophyll *a* and *b* content (mean ± standard deviation) in leaves of *S. procumbens* grown under greenhouse conditions in tailing and tailing/biochar substrates. Two-way ANOVA was performed to evaluate the effects of time (100 days) and treatment on leaf chlorophyll content, and simple regression analysis was used to assess the relationship between exposure time and chlorophyll content. Asterisks indicate significant differences between treatments at each exposure time (* *p* < 0.05, Tukey test). ANOVA test: *** = *p* < 0.001.

Additionally, regression analysis revealed a significant and negative relationship between exposure time and chlorophyll *a* and *b* concentrations in both treatments, indicating that chlorophyll levels in *S. procumbens* decreased as exposure time increased (Figure 4).

### 2.8. Effect of Tailing and Tailing/Biochar Substrates on Genetic Damage (Single-Strand DNA Breaks) of S. procumbens Individuals

**Figure 5:**
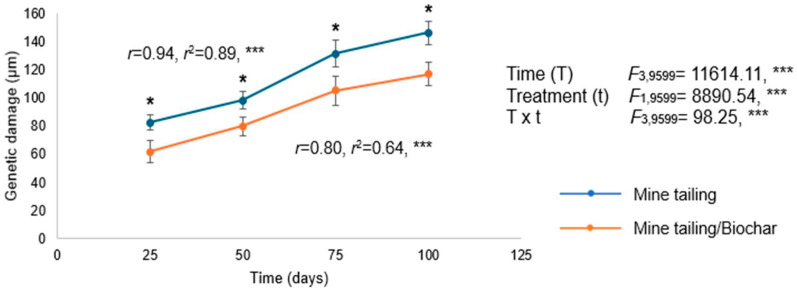
Genetic damage (mean ± standard deviation) in leaf tissue of *S. procumbens* grown under greenhouse conditions in tailing and tailing/biochar substrates. Two-way ANOVA was performed to evaluate the effects of time (100 days) and treatment on genetic damage, and simple regression analysis was used to assess the relationship between exposure time and genetic damage. Asterisks indicate significant differences between treatments at each exposure time (* *p* < 0.05, Tukey test). ANOVA test: *** = *p* < 0.001.

Our results showed a significant effect of time, treatment, and their interaction (time × treatment) on genetic damage (single DNA strand breaks) in the leaf tissue of *S. procumbens* individuals. Overall, we observed a statistically significant increase in genetic damage over the exposure period in plants grown in both substrates. However, individuals who grew in the tailing/biochar substrate exhibited significantly lower levels of genetic damage compared with those grown in the tailing substrate alone. Additionally, we found a positive and significant relationship between exposure time and genetic damage in both treatments, indicating that longer exposure periods were associated with higher levels of genetic damage (single-strand DNA breaks) in *S. procumbens* individuals (Figure 5).

## 3. Discussion

### 3.1. Physicochemical Characterization of Coconut Biochar and Its Influence on Heavy Metal Immobilization in Soil

The physicochemical properties of biochar are strongly related to its adsorptive potential for different pollutants and its applicability in the context of soil bioremediation. Among the most relevant are stability, pH, porosity, surface area, and the functional groups distributed on its surface [41]. Respect the pH, the typically presence of carbonates and phosphates in the biochar composition gives to biochar an alkaline profile, the pH is influenced by the characteristics of raw material employed in its production, as well as temperature of the pyrolysis process, higher temperatures rise the pH of the final biochar, a strongly alkaline profile indicates a high carbonization degree in the biochar, high alkaline pH favors the precipitation and immobilization of heavy metals via π-staking [42,43]. The coconut-fiber biochar evaluated in the present research has a strong alkaline pH of 10; due to this, the stabilization of heavy metals in mine-tailings could be related to heavy metal precipitation and immobilization. Similar strong alkaline pH characteristics have been reported in biochar produced from coconut fiber as a raw material. In the study of Sukartono et al. [44], the biochar showed a pH of 9.9, Devens et al. [45] characterized a biochar with a pH of 8.41 ± 0.25, while in the study of Guarnieri et al. [46], the biochar showed a pH of 9.03 ± 0.05, finally in the study of Lima et al. [47], the biochar produced revealed a pH of 9.56.

Under alkaline conditions, the bioavailability of HM tends to decrease [48]; thus, incorporating coconut fiber biochar into soils could contribute to reducing HM mobility and consequently their uptake by plants. Coconut fiber biochar contained a high proportion of OM (4.4%), which is known to affect HM mobility through complexation with ion-coordinating organic fractions and competition for sorption sites [49]. The observed OM content may therefore have contributed to HM immobilization, leading to reduced bioaccumulation in *S. procumbens* tissues. The nitrogen concentration was very low (0.033 mg·kg^−1^), a finding that is consistent with the naturally low N content reported for coconut fiber biochar (0.21–2 mg·kg^−1^; [33]). During pyrolysis, nitrogen losses are expected due to volatilization, which explains the reduced nitrogen levels found in this study. Additionally, a medium phosphorus content (10.5 mg·kg^−1^) was detected. Although this concentration is not high, the addition of biochar to soil substrates can provide a source of P available for plant uptake [50], which may favor plant growth, resulting in high biomass, a fact consistent with our results.

On the other hand, functional groups commonly present in biochar include several oxygen groups such as carboxyl, carbonyl, lactone, hydroxyl, and ketone groups, along with mineral crystals (like graphite). These components serve as active adsorption sites for heavy metals [51,52]. The pyrolysis process has a strong effect on the surface functional groups, mainly a reduction in the abundance of hydroxyl groups present in the pyrolyzed material, as has been observed in the FTIR characterization of biochar produced from different raw materials [53]. In the present study, the FTIR analysis revealed decreases in the intensity of the peaks associated with hydroxyl (–OH) and aliphatic C-H bonds stretching as a result of the pyrolysis process and the presence of carboxylic groups on biochar surface, similar findings were reported by Wu et al. [54], in biochar produced from coconut-fiber at 700 °C, as well as in the study by Suman and Gautam [55], in which the biochar was produced at temperatures between 400 and 1000 °C.

**Figure 6:**
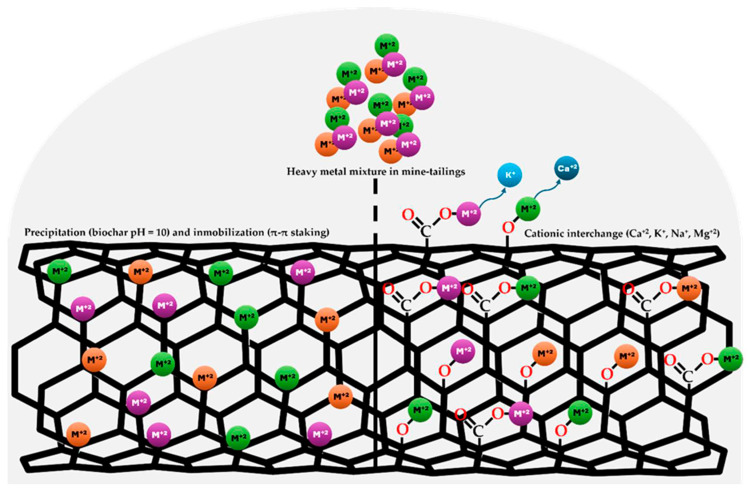
Implicated mechanisms in heavy metal stabilization in mine-tailings by coconut-fiber biochar.

In the case of divalent heavy metals such as Cd^+2^ and Pb^+2^ sorption in biochar are conducted through cation exchange and precipitation, interchangeable mineral elements linked to oxygen groups (carbonyl and carboxyl) in biochar include Calcium (Ca^+2^), potassium (K^+^), sodium (Na^+^), magnesium (Mg^+2^) Cation exchange and precipitation are important mechanisms for Cd and Pb sorption [56]. According to the composition of the coconut-fiber biochar evaluated in this study, the are several interchangeable elements such as Ca^+2^ (0.78%), K^+^ (0.17%), sodium (0.09%), and Mg^+2^ (0.02%), while the FTIR spectra of the biochar reveals the presence of surface carboxyl groups and a high carbonization degree in the biochar (Figure 1), according the pH, functional groups, composition and carbonization degree the heavy metal stabilization observed could be mediated by precipitation, immobilization and cationic interchange mechanisms (Figure 6).

Our results demonstrated that carbon (80%) and oxygen (18%) were the predominant elements in coconut fiber biochar. This finding aligns with earlier research indicating that carbon is the primary component of biochar, as the thermal decomposition of coconut fiber biomass during pyrolysis increases the elemental carbon fraction [33,57]. The dry weight yield of coconut fiber biochar was 31.6%. This result is supported by the distinctive decrease in biochar yield observed with increasing pyrolysis temperatures, primarily due to lignocellulose decomposition and the volatilization of OM [58]. Comparable reductions have been reported for coconut biochar, with yields ranging from 29.3% to 48.1% depending on pyrolysis temperature [9].

The TG analysis revealed a highly recalcitrant biochar. The increase in aromaticity at higher pyrolysis temperatures, as reported by Lee et al. [59], explains the recalcitrant nature of coconut shell biochar, which begins to decompose only above ~400 °C. This structural stability suggests a slow decomposition rate once applied to soils. Additionally, the formation of carboxyl groups at high pyrolysis temperatures has been associated with an enhanced cation exchange capacity [60], which could further reduce HM bioavailability.

Overall, the physicochemical properties of coconut fiber biochar—particularly its alkaline pH, OM content, and structural recalcitrance—indicate its potential for reducing HM mobility and bioavailability in soils, while also contributing to soil fertility through limited nutrient release, making coconut fiber biochar an ideal amendment for bioremediation strategies.

### 3.2. Influence of Coconut Fiber Biochar Incorporated in Tailing Substrate on HM Bioaccumulation, Bioconcentration and Translocation Factors in S. procumbens Individuals

*Sanvitalia procumbens* bioaccumulated various HMs (Zn > Pb > Cu > Cd) during 100 days of exposure. Overall, significant differences were observed among treatments across all exposure times. Individuals growing in tailing substrate with biochar exhibited significantly lower levels of HM bioaccumulation compared to those grown in tailings without biochar. Coconut fiber biochar has been reported as an effective amendment when used in combination with different plant species to phytoremediate soils contaminated by various HMs (Cd, Cu, Pb, and Zn), reducing their bioavailability and, consequently, lowering HM bioaccumulation compared to plants grown without coconut fiber biochar [6,26,61,62,63]. Another important finding was that individuals of *S. procumbens* grown in tailing substrate with biochar exhibited significantly lower BCF values in both roots and leaves, for all analyzed metals and across all exposure times, compared with individuals grown in tailings without biochar and the TF of *S. procumbens* for all HM in both treatments was <1, indicating that this species accumulates higher concentrations of metals in roots than in leaves, results that are in agreement with Ibrahim et al. [64] and Eissa [65]. These results may be attributed to the interaction of biochar with HMs, thereby reducing their bioavailability. In this regard, Xiao et al. [66], Zhang et al. [67], and Guo et al. [13] reported that biochar has various functional groups on its surface, which primarily interact with HMs through electrostatic interactions and ion exchange, leading to HM immobilization. In our study, coconut fiber biochar was found to contain carboxyl groups, which may serve as important sorption sites for HMs. Similar results were reported by Lu et al. [68] and Ahmad et al. [69], who demonstrated that carboxyl groups present in biochar derived from sewage sludge and peanut shells were responsible for forming complexes with HMs. These authors further noted that carboxyl groups can act as proton donors; therefore, deprotonated carboxyl groups may be involved in the coordination with HMs, mainly Pb and Cd. Moreover, the physicochemical characteristics of coconut fiber offer several advantages over other biomasses. For instance, coconut fiber biochar has been reported to exhibit hardness and resistance to degradation comparable to that of wood-derived biochar, due to its high lignin and cellulose content [70]. In addition, although coconut fiber is traditionally employed at an artisanal scale [71], its bioavailability is high, meaning that this feedstock is abundantly available and therefore does not compete for use as a biochar precursor—an essential criterion when selecting the biomass source for biochar production [10]. Finally, coconut fiber possesses a granular and porous structure, which provides higher pore density and enhanced water retention capacity, allowing it to retain up to nine times its weight in water [10]. Hence, the combination of plant species with biochar decreases the mobility of potentially toxic metals, thereby reducing their bioavailability, which in turn limits metal uptake by plants, mitigates the biomagnification of HM along the food chain, and decreases ecological and human health risks.

### 3.3. Influence of Coconut Fiber Biochar Incorporated in Tailing Substrate on Biomass Traits in S. procumbens Individuals

In general, individuals that grew in tailing substrate with biochar exhibited statistically higher biomass values compared to those grown in tailing substrate without biochar. Similar results have been reported assessing the effects of different biochars on biomass production in plant species [72,73,74,75,76], concluding that biochar has a positive effect on plant biomass production. Our results suggest that biochar can promote plant growth through multiple mechanisms, particularly through nutrient supply. For instance, coconut shell biochar contained a moderate concentration of phosphorus (10.5 mg·kg^−1^) and a high organic matter content (4.4%), indicating that its incorporation into tailing substrate may provide an additional nutrient source for plant uptake and thereby enhance biomass production. Moreover, it has been reported that biochar reduces the mobilization of HMs in soils and, consequently, their uptake by plants [74,75], which ultimately favors plant development and growth, resulting in increased biomass, an observation that supports our results. Different studies have highlighted the effectiveness of coconut fiber biochar as an amendment in the stabilization of heavy metals in polluted soils as an efficient alternative. Liu et al. [77] reported the use of coconut biochar (5%) to immobilize HMs (Cd, Ni, and Zn) in soils, reporting a bioavailability reduction of 30.1, 57.2 and 12.7%, respectively. Also, Li et al. [78] reported the use of coconut fiber biochar (2%) in the immobilization of Pb, while the use of this same biomass reduced Pb bioavailability in soils from 14 to 47%, and an increase in HM translocation to aerial tissues in *Oryza sativa* L [26].

### 3.4. Influence of Coconut Fiber Biochar Incorporated in Tailing Substrate on Chlorophyll a and b Content in S. procumbens Individuals

According to our results, individuals grown in tailings with biochar exhibited significantly higher contents of chlorophyll *a* and *b* at all exposure times compared to those individuals grown in tailings without biochar. These findings are consistent with previous studies, as Abid et al. [79] reported that tomato plants (*Solanum lycopersicum* L.) grown in Cd-contaminated soil showed increased levels of chlorophyll a, b, and total chlorophyll when biochar was applied. Similarly, Zeeshan et al. [80] found that tomato plants (*Lycopersicum esculentum* L.) displayed enhanced photosynthetic pigments, including chlorophyll *a* and *b*, following biochar supplementation. The mechanism by which biochar improves photosynthesis, particularly the production of chlorophyll *a* and *b*, may be explained by Lyu et al. [81], who indicate that biochar promotes the synthesis of proteins involved in electron transport, thereby positively influencing photosystem II activity and enhancing chlorophyll production in plants grown with biochar.

Furthermore, the reduction in HM bioaccumulation in leaves of plants grown with biochar was important for mitigating damage to chlorophyll *a* and *b* synthesis in *S. procumbens* individuals. In this regard, it has been reported that Cd can disrupt chlorophyll biosynthesis and degrade chlorophyll molecules, resulting in decreased levels of chlorophyll *a* and *b* [82]. Likewise, certain non-trace metals inhibit the uptake of essential nutrients, resulting in deficiencies and loss of function. For example, Pb^2+^ replaces Mg^2+^ in the porphyrin ring of the chlorophyll molecule, causing cells to accumulate protoporphyrin, and ultimately blocking chlorophyll biosynthesis [83,84]. On the other hand, Zn is a micronutrient; however, both its deficiency and excess can disrupt normal physiological processes in plants. In this context, Li et al. [85] reported that wheat plants exposed to high Zn concentrations exhibited several cellular abnormalities, including enlarged vacuoles, reduced cytoplasm, disordered basal lamellae in chloroplasts, mitochondrial membrane disintegration, and thickened cell walls, which impaired normal cell functions affecting chlorophyll *a* and *b* production. Altogether, these results demonstrate that metals have a negative impact on chlorophyll production and photosynthesis. In this context, biochar plays a key role in reducing their bioavailability, thereby decreasing their uptake by plants and mitigating their toxic effects, such as the inhibition of chlorophyll *a* and *b* synthesis, a fact that is very important for plants when using them for phytoremediation strategies.

### 3.5. Influence of Coconut Fiber Biochar Incorporated in Tailing Substrate on Genotoxic Damage (DNA Single Strand Breaks) in S. procumbens Individuals

In general, individuals grown in tailing substrate with biochar exhibited statistically lower levels of genotoxic damage (single-strand breaks) across all exposure times compared to those grown in tailing substrate without biochar. HM exposure leads to the generation of reactive oxygen species (ROS) within plant cells, which have the potential to damage macromolecules such as DNA, thereby causing oxidative damage [86]. Previous reports indicate that when plants bioaccumulate Cd and Pb, they show genotoxic damage, these elements are highly toxic, particularly at the cellular level, where they can induce genomic instability and exert both direct and indirect genotoxic effects, including DNA strand breaks, DNA–protein crosslinks, oxidative DNA damage, and inhibition of DNA repair enzymes [87,88], which may lead to DNA single strand breaks that are revealed in the alkaline comet assay. In this context, biochar, by reducing the bioaccumulation of toxic metals, positively contributed to lowering genotoxic damage levels in *S. procumbens* individuals grown in tailings amended with biochar. In this regard, Li et al. [89] evaluated the effect of maize-residue-derived biochar on tomato plants grown in quartz sand soil contaminated with HM. The results demonstrated that biochar-treated plants exhibited higher levels of antioxidant enzymes like superoxide dismutase (SOD), peroxidase (POD), and catalase (CAT). This enhancement was attributed either to the activation of enzymatic pools or to the overexpression of genes encoding these enzymes. Consequently, biochar has been shown to improve the antioxidant defense machinery, thus mitigating or reducing genotoxic damage. Similarly, Zulfiqar et al. [90] reported that wheat plants grown with straw-derived biochar exhibited increased activities of SOD, POD, and CAT compared with plants cultivated without biochar. The authors indicated that biochar application enhanced this antioxidant enzyme activity by improving plant metabolic functions and cell growth, as well as by reducing the accumulation of ROS. Hence, the induction of AP sites (apurinic/apyrimidinic sites), resulting from oxidized bases, that are evidenced as single strand breaks in the alkaline comet assay, may be reduced by adding biochar to accumulator plants. Finally, it is important to mention that this is the first study that reports the effect of coconut fiber biochar on reducing single-strand break induction in leaf tissue, a fact that is important when considering plant health for phytoremediation strategies.

## 4. Materials and Methods

### 4.1. Study Site

The study was conducted in Huautla, Tlaquiltenango Municipality, Morelos, Mexico, where approximately 780,000 metric tons of mine tailings rich in heavy metals (As, Cd, Cu, Cr, Fe, Mn, Pb, and Zn) have been reported [91]. Two primary tailings deposits (Tailing 1: 18°26′07.7″ N, 99°01′16.9″ W; Tailing 2: 18°26′20.9″ N, 99°01′54.9″ W) are located approximately 500 m and 1000 m from the main settlement, respectively. Both deposits exhibit similar physicochemical properties, with pH ranging from 7.85 to 8.37, electrical conductivity from 0.2 to 0.4 dS m^−1^, organic matter (OM) content from 0.52 to 0.84%, particle size < 45 μm, and an elevation of 974 m above sea level [92]. The bioavailable concentrations (mg·kg^−1^) of heavy metals in the tailing’s substrate were Cu (8), Cd (8), Pb (46), and Zn (428) [25,93]. Tailings samples were collected at three depths: surface, mid-layer, and base, by obtaining five subsamples per depth, at 10 m intervals along a 50 m linear transect. Sampling was conducted from the surface to a depth of 30 cm, and subsamples from each tailings deposit were homogenized to obtain a representative sample for each site. Heavy metal concentrations (mg·kg^−1^) were determined using atomic absorption spectrophotometry with the flame method (GBC 908 A, GBC Scientific Equipment Pty Ltd., Keysborough, Australia).

### 4.2. Study Species

*Sanvitalia procumbens* Lam. (Asteraceae) is an annual, herbaceous therophyte exhibiting either a procumbent or erect habit, occasionally forming dense, mat-like aggregations. Individuals attain up to 50 cm in height and 80 cm in lateral spread. Leaves are opposite, narrowly elliptic to lanceolate, entire-margined, and measure up to 5 cm in length. The capitula are radiate, comprising yellow–orange ligulate florets and a dark brown disk of tubular florets, morphologically reminiscent of *Helianthus annuus* [94]. The species is native to Mexico and Central America, with a broad ecological amplitude in Mexico, where it occurs in tropical deciduous forests, pine–oak woodlands, xerophytic scrub, and natural grasslands. Its altitude extends from 8 to 2750 m [95].

### 4.3. Seed Germination and Seedling Establishment

Mature seeds of *S. procumbens* were collected from 50 individuals at Quilamula, Tlaquiltenango, Morelos, Mexico (18°30′52″ N, 95°59′59″ W; 1020 m a.s.l.), according to Gold et al. [96]. This site was chosen because it has similar environmental and climatic conditions to Huautla mine tailings but no history of mining activity. Seeds were stored in labeled kraft paper bags and cleaned in the laboratory. Visually healthy seeds were selected, excluding empty or parasitized ones, and germinated in Petri dishes on Whatman^®^ filter paper moistened daily with 5 mL KNO_3_. After radicle emergence, seedlings were grown in peat moss (200 mL pots) until the first true leaves appeared (~15 days), then transplanted into 1 L pots with either mine tailing-agrolite substrate (75:25, *v*/*v*) or a 75:25 (*v*/*v*) tailing–biochar mix (*n* = 90 per treatment). Agrolite is a specific type of perlite used as a soil amendment. It is a sterile, porous material that acts as a neutral substrate. Biochar/tailing proportions were selected according to Rosas-Ramírez et al. [25]. Biochar was ground and sieved to 0.5 mm to match the tailings particle size with a stainless-steel sieve (Fiicsa, Mexico City, Mexico). Plants were maintained in a greenhouse at 32–35 °C with regular irrigation. Sampling occurred every 25 days for a 100-day period. We analyzed HM bioaccumulation (roots and leaves), biomass production, genotoxicity (single-strand DNA breaks), and chlorophyll *a* and *b* content in 12 plants per treatment.

### 4.4. Coconut Fiber Biochar Production and Physico-Chemical Characterization

Coconut fiber biochar was produced using agro-industrial waste as raw material. Coconut fiber was pyrolyzed at 600 °C. The process yield and dry weight (D.W.) were calculated by the ratio between the mass of dry biochar obtained after the carbonization process and the initial dry mass of coconut fiber, expressed as a percentage. Subsequently, biochar was physiochemically characterized through the determination of the following parameters: pH, organic matter percentage, organic carbon, total phosphorus, and total nitrogen, according to the procedures established in the Mexican standard [97]. The elemental analysis was conducted through Field Emission Scanning Electron Microscopy with Energy Dispersive X-Ray Spectroscopy (FE-SEM-EDS; JEOL JSM-7800F/Oxford Instruments X-Max 80 detector, Boston, MA, USA). To improve conductivity, the samples were covered with gold by cathodic sputtering prior to determinations. The organic composition of the coconut fiber biochar was determined through a PerkinElmer 2400 Series II CHNS/O elemental analyzer (Shelton, CT, USA); the content of carbon, hydrogen, nitrogen and sulfur was determined under standard conditions and calibrated with L-cysteine as a reference standard.

The characterization of the functional groups present on the surface of the coconut fiber biochar was carried out by Fourier transform infrared spectroscopy (FTIR), using an Infrared Spectrophotometer FTIR-7600 from Lambda Scientific Pty Ltd. Before determination, biochar samples were oven-dried (60 °C; 12h) to eliminate the sample moisture. Subsequently, biochar pellets were prepared using the KBr dispersion method. Measurements were performed in a spectral range of 4000 to 400 cm^−1^, with a resolution of 4 cm^−1^, accumulating 32 scans per sample to improve the signal-to-noise ratio. This analysis allowed the identification of the main oxygenated and aromatic functional groups derived from the original lignocellulose precursor, as well as those generated or modified during the carbonization process of the original material. Finally, the thermal degradation profiles of the coconut fiber biochar were obtained by thermogravimetric analysis (TGA) under a N_2_ flow (50 mL/min), heating at 20 °C/min up to 600 °C, in a TA Instrument (Model SDT Q600).

### 4.5. Biomass Determination

The effect of biochar on plant growth was assessed by measuring physiological and biomass traits, including root and leaf wet weight and root and leaf dry weight (g).

### 4.6. Heavy Metal Concentrations

Three coconut fiber biochar samples were analyzed to determine metal concentrations (Cd, Cu, Pb, and Zn). Every 25 days, 12 individuals of *S. procumbens* growing in mine tailings and 12 growing in mine tailings/biochar samples were collected to quantify Cd, Cu, Pb, and Zn concentrations in root and leaf tissues. Root and leaf tissues were separated and washed three times with distilled water to remove substrate residues. Clean tissues were oven-dried at 60 °C for 72 h to constant weight. Subsequently, samples were ground, and 0.25 g of dry tissue from each structure was weighed and placed in closed Teflon bombs for digestion with 10 mL of HNO_3_ (70%) using a microwave-assisted reaction system (CEM MARS-5, Matthews, NC, USA). Digested samples were diluted and filtered with distilled water to a final volume of 50 mL. A blank (without tissue) was processed simultaneously as a negative control. Metal concentrations were determined by atomic absorption spectrophotometry using the flame method (GBC 908 A, GBC Scientific Equipment Pty Ltd., Keysborough, Australia). Calibration was performed with standard solutions of known concentrations prepared from pure metal ions (ULTRA Scientific, North Kingstown, RI, USA). All calibration curves exhibited correlation coefficients (R^2^) ranging from 0.995 to 1.0. The detection limits (mg·kg^−1^) of the atomic absorption spectrophotometer were Cd (0.0004), Cu (0.001), Pb (0.001), and Zn (0.0005). For each sample, metal concentrations were determined in triplicate and reported as mean values in mg·kg^−1^.

### 4.7. Bioconcentration Factor (BCF) and Translocation Factor (TF)

The phytoextraction capacity of *S. procumbens* was evaluated using two indexes: (1) the bioconcentration factor (BCF), which measures the efficiency of the plant to accumulate metals from the substrate into its tissues, and (2) the translocation factor (TF), which quantifies the efficiency of metal transport from roots to shoots [98]. These indexes were calculated as follows:

$${BCF}_{root}=\frac{C_{root}}{C_{tailing}}$$

$${BCF}_{leaf}=\frac{C_{leaf}}{C_{tailing}}$$

where C_leaf_ is the metal concentration in leaf tissue, C_root_ is the metal concentration in root tissue, and C_tailing_ is the bioavailable metal concentration in mine tailings.

A plant species is considered a phytoextractor when TF > 1, and a phytostabilizer when TF < 1 for the analyzed metal [98,99]. A Student’s *t*-test was performed to determine whether significant differences existed between treatments (tailings and tailings/biochar) in BCF and TF values for Cu, Cd, Pb, and Zn.

Heavy metal immobilization percentage is calculated as the difference between the metal concentration in the reference treatment (mine tailing without biochar) and the treated sample (mine tailing/biochar), relative to the reference treatment.

### 4.8. Single Cell Gel Electrophoresis or Comet Assay for Leaf Tissue

Genetic damage was assessed at each sampling time by randomly selecting 24 individuals (12 per treatment: tailings substrate and 75% tailing + 25% biochar substrate). The assay was performed following the protocol of Tice et al. [100], with minor modifications. Leaves were rinsed with distilled water to remove substrate residues. The leaves were then placed in glass Petri dishes containing 2 mL of cold phosphate-buffered saline (PBS, pH 7.5, 4 °C). For nuclei isolation, leaf tissues were cut with a razor blade and kept on ice. Microscope slides were pre-coated with 125 µL of 1% normal-melting agarose and dried at 60 °C. Subsequently, 50 µL of cell suspension was mixed with 50 µL of 1% low-melting-point agarose in an Eppendorf tube. From this mixture, 80 µL was pipetted onto the pre-coated slides, covered with a coverslip, and placed on ice for five min. After agarose solidification, coverslips were removed, and a final layer of 0.5% low-melting-point agarose was added. Once solidified, coverslips were removed. Slides were immersed for 24 h at 4 °C in lysis solution (2.5 M NaCl, 100 mM EDTA, 10 mM Tris, pH 10, 10% DMSO, 1% Triton X-100, and 1% SDS). Gels were then transferred to an electrophoresis chamber, covered with cold alkaline buffer (300 mM NaOH and 1 mM EDTA, pH 13.0) for 20 min to allow DNA unwinding. Electrophoresis was carried out at 300 mA and 25 V for 20 min under dark conditions. After electrophoresis, slides were neutralized with three washes of Tris buffer (0.4 M, pH 7.5), five min each, and subsequently dehydrated with cold ethanol (96%) for fixation. Finally, slides were stained with 20 µL of ethidium bromide solution (15 µg/mL) and visualized using a fluorescence microscope (Carl Zeiss, Jena, Germany) equipped with an excitation filter (515–560 nm) and a barrier filter (590 nm). For each individual, 100 cells were analyzed. DNA migration was quantified by measuring comet tail length (µm).

### 4.9. Chlorophyll a and b Content

Chlorophyll *a* and *b* were quantified at 25, 50, 75, and 100 days using a chlorophyll meter (ClorofiLOG CFL 1030, Porto Alegre, Brazil). Values were expressed as the Falker Chlorophyll Index (FCI), based on photodiode emissions at 635, 660, and 880 nm. For each treatment (tailing and tailing/biochar), 24 individuals were randomly selected, and three leaves from the lower, middle, and upper thirds of each plant were measured. All readings were taken at the center of the leaf blade between 9:00 and 10:00 a.m.

### 4.10. Statistical Analysis

Data normality was assessed with the Shapiro–Wilk test. Two-way ANOVAs were used to evaluate the effects of substrate (tailing and tailing/biochar), exposure time (25, 50, 75, 100 days), and their interaction on biomass, chlorophyll content, and genetic damage in *S. procumbens*. Tukey’s post hoc test (*p* < 0.05) was applied to identify pairwise differences, and simple regressions were performed to examine relationships between exposure time and each analyzed trait. A separate two-way ANOVA was conducted to test the effects of exposure time, treatment, and their interaction on Cu, Cd, Pb, and Zn bioaccumulation in roots and leaves, followed by Tukey’s test to compare mean metal concentrations among treatments [101]. All analyses were conducted in STATISTICA 8.0 [102].

## 5. Conclusions

The results of this study demonstrate that *S. procumbens* can bioaccumulate Cd, Cu, Pb, and Zn in root and leaf tissues. The physicochemical properties of coconut fiber biochar were strongly related to its adsorptive potential for HM and its applicability in soil bioremediation. Among the most relevant were stability, pH, porosity, surface area, and the functional groups distributed on its surface. The addition of coconut fiber biochar as an amendment further improves the system by reducing HM bioaccumulation, decreasing genetic damage, enhancing biomass production, and increasing chlorophyll content. Hence, assisted phytoremediation by incorporating coconut fiber biochar in combination with *S. procumbens* strengthens phytostabilization by reducing HM biomagnification within the trophic chain and mitigating the negative impacts of HM exposure on ecosystem health.

## Figures and Tables

**Table 1:** Bioconcentration factor (BCF) and translocation factor (TF) of Cu, Cd, Pb, and Zn (mean ± standard deviation) in roots and leaves of *S. procumbens* grown under greenhouse conditions. Student *t*-tests indicate significant differences between treatments by plant structure (degrees of freedom = 95 in all cases). *** = *p* < 0.0001.

Concentration (mg·kg^−1^)							
Metal	Time (Days)	BCF (Root)		BCF (Leaf)		TF	TF
		Tailing	Tailing/Biochar	Tailing	Tailing/Biochar	Tailing	Tailing/Biochar
Zinc							
	25	1.76	1.13	1.05	0.42	0.60	0.37
	50	1.94	0.98	0.86	0.30	0.44	0.34
	75	2.58	0.61	0.69	0.28	0.26	0.47
	100	2.68	0.41	0.63	0.27	0.23	0.67
	mean ± SD	2.24 ± 0.46	0.76 ± 0.32	0.81 ± 0.18	0.32 ± 0.07	0.39 ± 0.16	0.50 ± 0.20
	*t*-student	32.195 ***		24.938 ***		4.407 ***	
Copper							
	25	9.01	5.23	4.31	3.09	0.47	0.59
	50	10.79	3.53	5.53	2.58	0.51	0.73
	75	13.85	3.57	6.49	2.77	0.46	0.77
	100	15.09	3.39	7.52	2.76	0.50	0.81
	mean ± SD	13.17 ± 2.76	3.93 ± 0.86	5.96 ± 1.36	2.80 ± 0.20	0.49 ± 0.07	0.76 ± 0.21
	*t*-student	32.656 ***		28.167 ***		12.486 ***	
Lead							
	25	49.70	18.81	36.22	11.40	0.39	0.54
	50	51.97	15.82	15.92	5.97	0.43	0.48
	75	55.72	19.34	21.29	7.61	0.42	0.39
	100	67.27	20.23	22.55	6.89	0.37	0.34
	mean ± SD	56.17 ± 7.81	18.54 ± 3.16	23.99 ± 8.64	7.97 ± 2.38	0.40 ± 0.05	0.45 ± 0.12
	*t*-student	53.747 ***		19.893 ***		3.495 ***	
Cadmium							
	25	2.88	0.97	0.00	0.00	0.00	0.00
	50	4.66	2.33	0.74	0.00	0.15	0.00
	75	5.76	3.09	1.87	0.79	0.32	0.26
	100	6.47	3.01	2.04	0.76	0.31	0.25
	mean ± SD	4.94 ± 1.46	2.33 ± 0.91	1.16 ± 0.86	0.38 ± 0.39	0.20 ± 0.15	0.12 ± 0.14
	*t*-student	18.061 ***		9.837 ***		4.484 ***	

## Data Availability

The original contributions presented in this study are included in the article/Supplementary Material. Further inquiries can be directed to the corresponding authors.

## Supplementary Materials

The following supporting information can be downloaded at: https://www.mdpi.com/article/10.3390/plants14223470/s1. Figure S1. FTIR spectrum of the coconut-fiber biochar. Dash red lines indicate the absence of the representative bands of hydroxyl and aliphatic C–H bonds. Blue lines indicate the corresponding bands of the chemical functional groups observed in the analysis. Colors indicate the areas covered for the bands. Green: hydroxyls; light blue: aliphatic C–H bonds; orange: aromatic C=O; dark green: C–O in cellulose residues; magenta: aromatic C–H bonds; and dark blue: C–H bonds as a result of lignin carbonization. Figure S2. TGA profile of the coconut fiber biochar under nitrogen atmosphere.
