# Supplementary figures and images for: Phytoremediation Potential of Heavy Metals Using Biochar and Accumulator Plants: A Sustainable Approach Towards Cleaner Environments
Source: Plants (Basel). 2025 Nov 14;14(22):3470. doi: 10.3390/plants14223470 (PMC12656689; doi:10.3390/plants14223470)

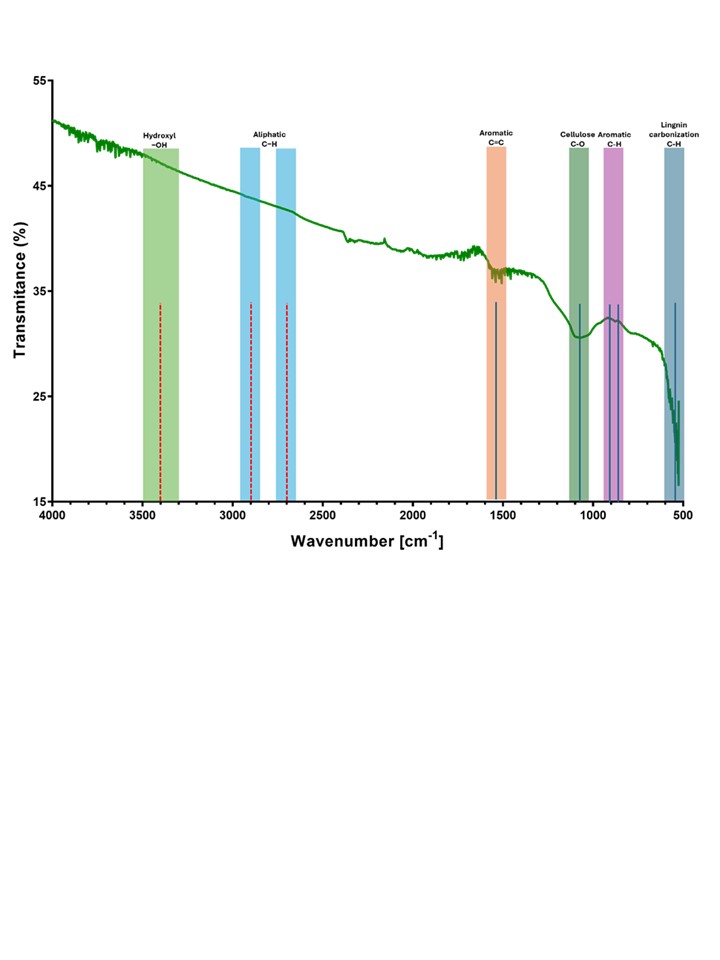

Supplement: Supplementary file 1 [file plants-14-03470-s001.zip › Figure S1.jpg]

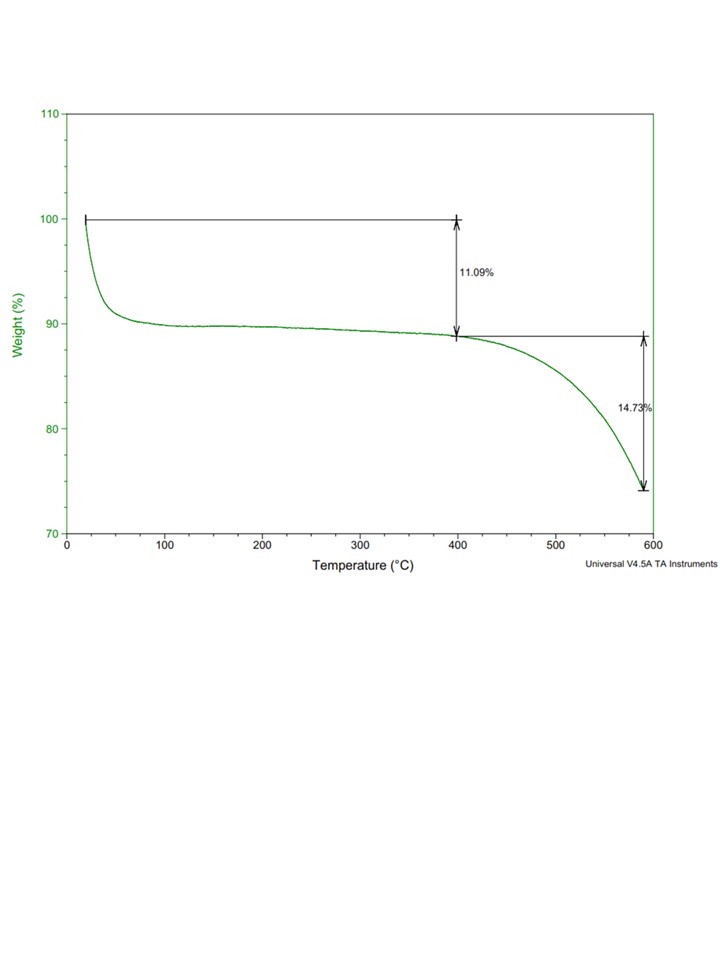

Supplement: Supplementary file 1 [file plants-14-03470-s001.zip › Figure S2.jpg]
